# The City of London

**Published:** 1914-08-15

**Authors:** 


### London Hospitals: Departures and Plans.

### THE CITY OF LONDON.

The City Branch of the British Red Cross Society have issued an appeal through the Lady Mayoress for £5,000 to make their Voluntary Aid Detachments exceptionally efficient. A further appeal is made for the loan of suitable halls and buildings within the City available for use as Temporary hospitals. The War Office has commandeered Bancroft's School, Woodford Wells, the property of the Drapers' Company, for use as a military hospital. The Corporation of London have agreed for the use of the Guildhall as a centre for the Voluntary Aid Detachments of the City, and as a depôt for the stores and equipment necessary. They have further agreed, should occasion arise, to fit out the hall as a Temporary hospital for the sick and wounded. Lady Wynne has undertaken to receive and classify the stores, which will be kept in the crypt of the Guildhall. The *personnel* of the City Detachments amounts to nearly 500.

### St. Thomas's Hospital and the Crisis.

The house officers at St. Thomas's Hospital are appointed for periods of six months. At the outbreak of the war a large number of men were about to finish their appointments. The authorities gladly made arrangements to fill their places for the last fortnight, so that they were free to volunteer for service in the Army or Navy. Over twenty men from St. Thomas's were at once accepted, and have been appointed to important billets, some going to sea, some going to Base hospitals as far as the Navy is concerned, some being placed in hospital, and others to Field Ambulance as far as the Army work is concerned.

The number of clerks and dressers in the wards has been seriously depleted by the call to the Territorials, the St. Thomas's students for a long time having been encouraged to train either in the R.A.M.C. or the Territorials, and several have thus been called upon as holders of commissions and others to serve as rankers. All have gone off with cheerful spirits to serve their country. Many of the senior staff of St. Thomas's, together with the senior staff of Guy's and the London Hospital, are gazetted for services on No. 2 Territorial Hospital, and many of the St. Thomas's R.A.M.C. students will act as dressers and orderlies in the Territorial No. 2 Hospital, which is being organised at St. Mark's College, Chelsea.

The main work of St. Thomas's, however, in this time of stress will lie within its own walls. Two hundred beds have been placed at the immediate disposal of the public Services—more to be provided as needed—and this offer has been communicated both to the Navy and Army. The Admiralty and the War Office have accepted most cordially this offer.

The wards of St. Thomas's Hospital afford good accommodation for the general number of patients, and can well be made to hold more at a pinch. Five wards can be prepared at any moment, and will be ready at a few hours' notice for the reception of at least 200 patients—soldiers or sailors—if needed. The governors are prepared to provide more beds as necessity arises, and to tax the resources of the hospital to the utmost limit. In this movement the staff most heartily join, and have cordially and willingly given all their services. None have gone away to any distance for a holiday, in spite of the hard work in the past session, and are within easy call.

The organisation of operating theatres, $x$-ray department, research laboratories, and physical exercise department, are thus at the service of our soldiers and sailors. Any extra demands on the staff can easily be met, as many old St. Thomas's men have willingly volunteered to come forward to meet the pressure.

St. Thomas's is fortunate inasmuch as it has now in stock an ample supply of surgical and medical necessaries to meet any grave emergency. Throughout the few days of crisis the hospital contractors nobly stood by the institution, and strove to keep up the supply, the only hitch being in regard to one large firm, who had to announce that, all their horses being commandeered by the Government, they could not deliver. The difficulty was readily met by the hospital authorities sending for the provisions and fetching them back in taxi-cabs. Many friends of the hospital have come forward and offered aid. Lady Henry Somerset has kindly placed her excellent hospital at Reigate at the disposal of the St. Thomas's authorities. Directly the call is made, this and other places will be immediately used by the transference to it of sad and serious cases which cannot be discharged to their homes, but not such acute cases that they need to be retained in the hospital, and thus the hospital beds other than those so needed for the treatment of acute cases will be free for the reception of our sick or wounded soldiers and sailors for hospital treatment. This means a very serious increase in the hospital work, and it is hoped that the friends of the hospital will rally round and ensure so excellent a scheme being fully carried out.

### The Duchess of Devonshire's Action.

The Duke and Duchess of Devonshire have placed a considerable portion of Devonshire House at the disposal of the British Red Cross Society for the purposes of a hospital and as a centre for this Society's work. A call for doctors, nurses, dressers, and orderlies has been made by this Society, who are also anxious to obtain offers of private houses. The Duke of Sutherland, Lord Desborough, Lord Spencer, the Archbishop of Canterbury, and many others have already offered their houses for the purpose.

